# Speech emotion classification using attention based network and regularized feature selection

**DOI:** 10.1038/s41598-023-38868-2

**Published:** 2023-07-25

**Authors:** Samson Akinpelu, Serestina Viriri

**Affiliations:** School of Mathematics, Statistics and Computer Science, University of KwaZulu-Natal, Durban, 4000 South Africa

**Keywords:** Mathematics and computing, Computational science, Computer science

## Abstract

Speech emotion classification (SEC) has gained the utmost height and occupied a conspicuous position within the research community in recent times. Its vital role in Human–Computer Interaction (HCI) and affective computing cannot be overemphasized. Many primitive algorithmic solutions and deep neural network (DNN) models have been proposed for efficient recognition of emotion from speech however, the suitability of these methods to accurately classify emotion from speech with multi-lingual background and other factors that impede efficient classification of emotion is still demanding critical consideration. This study proposed an attention-based network with a pre-trained convolutional neural network and regularized neighbourhood component analysis (RNCA) feature selection techniques for improved classification of speech emotion. The attention model has proven to be successful in many sequence-based and time-series tasks. An extensive experiment was carried out using three major classifiers (SVM, MLP and Random Forest) on a publicly available TESS (Toronto English Speech Sentence) dataset. The result of our proposed model (Attention-based DCNN+RNCA+RF) achieved 97.8% classification accuracy and yielded a 3.27% improved performance, which outperforms state-of-the-art SEC approaches. Our model evaluation revealed the consistency of attention mechanism and feature selection with human behavioural patterns in classifying emotion from auditory speech.

## Introduction

Human has various ways of exhibiting their emotion, which has placed them at the highest level of civilization among other creatures. These expressions can take the form of speech, facial, gestures, and other physiological modes. However, interaction and relationships among individuals are best sustained through communication from human speech. Human speech carries huge para-linguistic^1^ content that can reveal the state of emotion, both in direct and indirect communication. Therefore, speech emotion classification has been occupying a key position in advancing affective computing and speech research domain. Besides, unlike other methods of recognizing emotion, speech emotion can be said to reveal 90% of the intent of the speaker without pretence, hence, the reason why it is sporadically attracting researchers within the last decade.

In SEC, the cultural and racial background may have a significant impact, but the ground truth remains that emotion is universal. Because of peculiarities associated with the speech emotion domain, efforts have been made by professionals to generate a standardized synthetic dataset (emotional corpus) that had been useful for conducting research on emotion classification^2^. Among these corpora are IEMOCAP (Interactive and Diadic Motion Capture), TESS (Toronto English Speech Set), RAVDESS (Rayson Visual Emotion Speech Set), EMOVO, etc and their performances concerning speech emotion classification has been yielding appreciable result, even when sometimes compared with real world dataset. These datasets came in different languages (English, Spanish, German, Chinese)^3^. Speech emotion classification has its application in customer support management, self-driving cars, psycho-medicine, e-learning, etc. Its importance in human-computer interaction cannot be overemphasized. Gordon^4^ opined that affective behaviour may serve as a precursor to the emergence of mental health conditions like depression and cognitive decline and may aid in the development of therapeutic tools for automatically identifying and tracking the progress of diseases.

**Figure 1:**
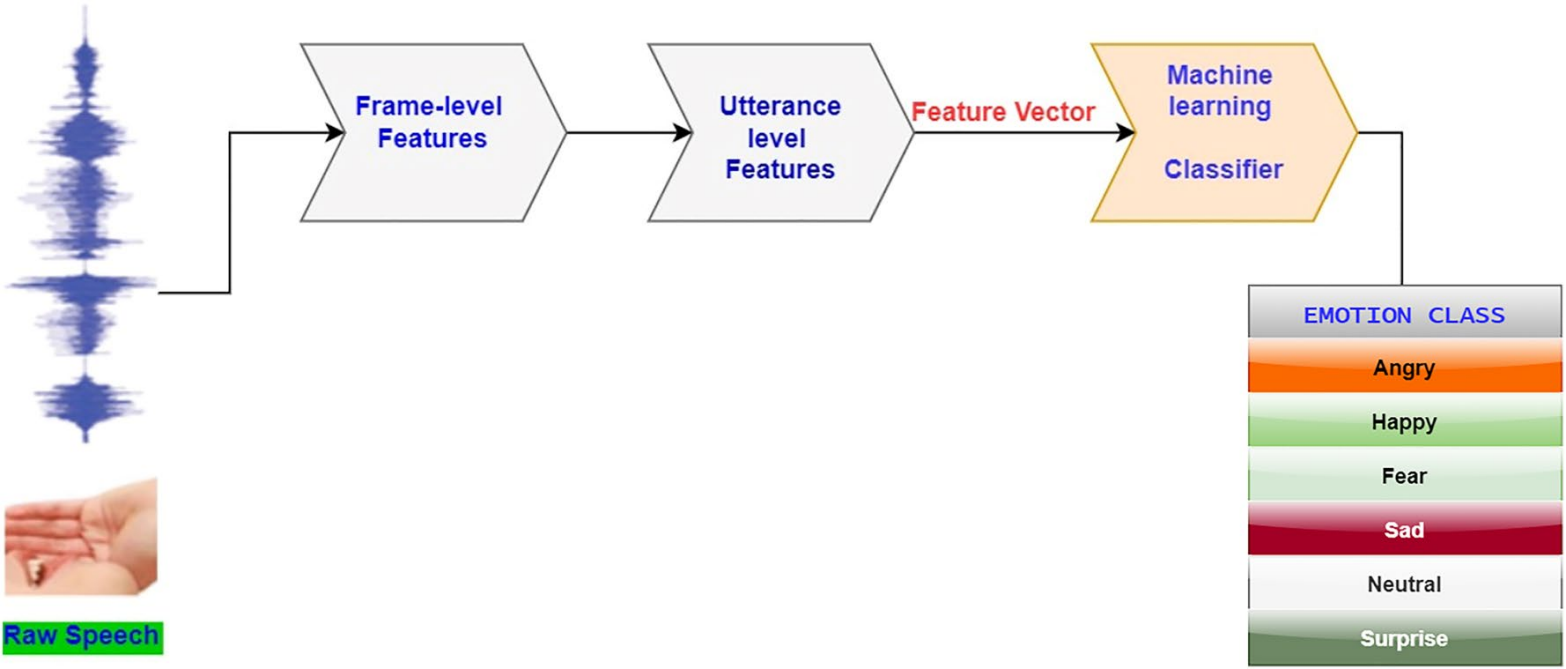
Conventional speech emotion classification system.

Classical techniques of classifying emotion in the past follows the extraction of primitives, acoustic features and low-level detectors (LLD), from raw speech^5^. These features (pitch, energy, etc) represents frame-level features and speech analysis on it, do generate another level of features (Utterance-level). Thereafter, the concatenation of these feature in vector form will be fed into a machine learning algorithm also referred to as classifiers in this context, for actual classification of emotion. Support Vector Machine (SVM), Gaussian Mixture Model (GMM), Hidden Makov Model (HMM) and K-Nearest Neighbour (KNN) are popular classifiers^6–8^. Figure 1 shows a classical structure of emotion recognition.

Though these approaches have proven to be efficient in their capacity, however, they are bewildered with salient challenges that rendered them unsuitable in achieving state-of-the-art result for SEC.

The focus of this study is to enhance and improve performance of speech emotion classification through attention-based network and feature selection techniques. To the best of our knowledge, this is the first-time feature selection is to be fused with attention layer of high dimensional features extracted from deep convolutional neural network, for accurate emotion classification (Attention based DCNN+RNCA+RF). We utilized TESS dataset in this study as a standard speech emotion corpus which captures seven classes of emotions express by human. The main contributions of this study are:

1. To experiment the efficiency of attention mechanism and regularized feature selection (Regularized Neighbourhood Component Analysis) techniques for speech emotion classification. A pretrained transfer learning network is set up as the based model. The feature selection neutralizes additional parameter weight added by attention layer and thereby minimize complexity.
2. To propose an Attention-based DCNN+RNCA+RF. After exploratory and thorough experiment with three different classifiers, our model achieved 97.8% accuracy on TESS dataset.

The remainder of the article is arranged as follows. An overview of related works is presented in Section “Review of related works”. The proposed technique and methods are described in Sect. “Methods and techniques”. Results and discussions are given in Sect. "Experimental results and discussion", while Sect. "Conclusion" is the conclusion and future recommendation for further study.

## Review of related works

The classification of emotion has its history traced to psychological submission^9,10^ where human emotion are grouped into six main classes (Sadness, Happiness, Anger, Disgust, Surprise and Fear). However, affective computing cannot be based on this primitive divisions, as computers are not perceiving moods, but they are interpreting them as a set of sequence of technical parameters, that are captured from the audio decoding process. Therefore, speech emotion classification requires efficient learning of paralinguistic information that can mitigates misclassification of emotion. The machine learning classifiers were first explored for SEC before the application of convolutional neural network models. The shortcomings of conventional classification approaches have paved ways for Convolutional Neural Networks (CNNs)^11,12^ and LSTM networks^13^. Occasionally, these two combined to form a robust model^14^ which have been widely employed in sequence modelling and its associated domain. A feature selection-based CNN was utilized by Farooq et al.^15^, for combating the artificial design influence which hampered accurate description of speakers’ emotional condition. Hajarolasvadi & Demirel^16^ proposed 3D-CNN for speech emotion classification based on overlapping frames segregation and MFCC features extraction. A 10-fold cross-validation parameter was used in their evaluation on three publicly available speech corpora, which were Ryerson Multimedia Laboratory (RML), Survey Audio-Visual Expressed Emotion (SAVEE) and eNTERFACE’05. The convolutional model achieved 81.05% accuracy on six emotions classes. Deep Belief Network (DBN) and SVM was proposed in Zhu et al.^17^ for extracting acoustics features, MFCC and zero-crossing rate were employed before emotion classification. Wang et al.^18^ combined Deep Neural Network and Extreme Learning machine (ELM) for speech emotion classification through the encoding of speech features (pitch and formants) and segmentation of audio feature vectors.

However, conventional CNN performs woefully in high-dimensional speech features extraction. This, and many more shortcomings, paved the way for the introduction of recurrent neural network (RNN) model. It was a great milestone improvement over CNN in speech emotion classification, because it addresses the failure of CNN in time-series data extraction. RNN has a hidden layer in its structure that updates the output value with respect to time on constant basis^19^. Kerkeni et al.^20^ proposed a RNN for speech emotion classification through analysis of speech signal using Teager-Kaiser Energy Operator (TKEO) combine with empirical mode decomposition (EMD). After extraction of speech cepstral features, SVM classifier was utilized for multi-classification of emotion. They achieved 91.16% on Berlin and Spanish based dataset. Nevertheless, RNN also suffers from dependency (long-term) and gradient descent problems. In some studies, CNN and RNN were combined to form a hybrid CRNN (Convolution Recurrent Neural Network) model to enhance speech emotion classification^21^.

As RNN is not isolated from its own limitations and by way of proffering quick fix to the issues peculiar to it, Long-Short-Term-Memory (LSTM) was proposed by Hochreiter & Schmidhuber^22^ and its combination with convolutional neural network has yielded a notable improvement. LSTM is a variant of RNN consisting of feedback connections for dependency learning in sequence prediction. A 1D and 2D CNN was combined with LSTM for SEC, which resulted in an appreciable accuracy of 82.4% with EMO-DB speech corpus by Zhao et al.^23^. Puri et al.^24^, proposed a hybridized LSTM, CNN and DNN approach for speech emotion classification. MFCC and mel-spectrogram were fed into eight contiguous 2D convolutional sequential neural network layers of their model. RAVDESS dataset was used, but there was no accuracy of emotion recognition reported. Besides, their technique is expensive to train because of the huge convolutional layers adopted. LSTM has a key component called forget gate and research has proven that it has high probability of forgetting emotional feature, while it focuses on the most recent ones and this hampered its efficiency within SEC domain.

Recent advancement in deep learning coupled with incessant search for a way of improvement and addressing the age long challenges in SEC made Bahdanau et al.^25^, to introduce attention network which is able to sieve out irrelevant information peculiar to speech data and concentrate on emotional rich information. Attention mechanism has been successfully adapted to other object recognition discipline with a notable improvement in models’ performance. An attention-based network was adopted in the work of Qamhan et al.^26^, where an accuracy of over 60% was achieved on IEMOCAP dataset. Attention models emulate the human way of focusing on important features for the recognition of an object.

Three-dimensional attention-based CRNN was used by Chen et al.^27^ to choose discriminative features for speech emotion classification. Their proposed model’s input layer accepted a Mel-spectrogram with delta-deltas. The employed delta-deltas reduced the intrusion of unimportant elements that can result in subpar classification performance, while keeping vital emotional data. Finally, a mechanism for attention that could take salient aspects into account was adopted. With an accuracy report of 82.82% on EMO-DB and 64.74% on the IEMOCAP speech dataset, their experiment’s outcome was supported the efficacy of attention technique for emotion classification.

Zhao et al.^28^. utilized attention-based model comprises Bidirectional LSTM, a Fully Connected Networks (FCN) for learning spatio-temporal emotional features and machine learning classifier for speech emotion classification. In the same vain, the author in Du et al.^29^. utilized attention-based model and 1Dimensional CNN for SEC. Softmax activation function was used at the top layer of their model after feature extraction. A cross-modal SEC was carried out in Seo and Kim^30^ using Visual Attention Convolutional Neural Network (VACNN) in partitioning the spectral feature from dataset. Combining speech dataset with text and video requires special techniques in extracting features for efficient prediction of emotion. In Zhang et al.^31^, the author applied 5 attention heads mechanism for multimodal speech emotion classification. Their novel model achieved 75.6% on IEMOCAP dataset.

**Figure 2:**
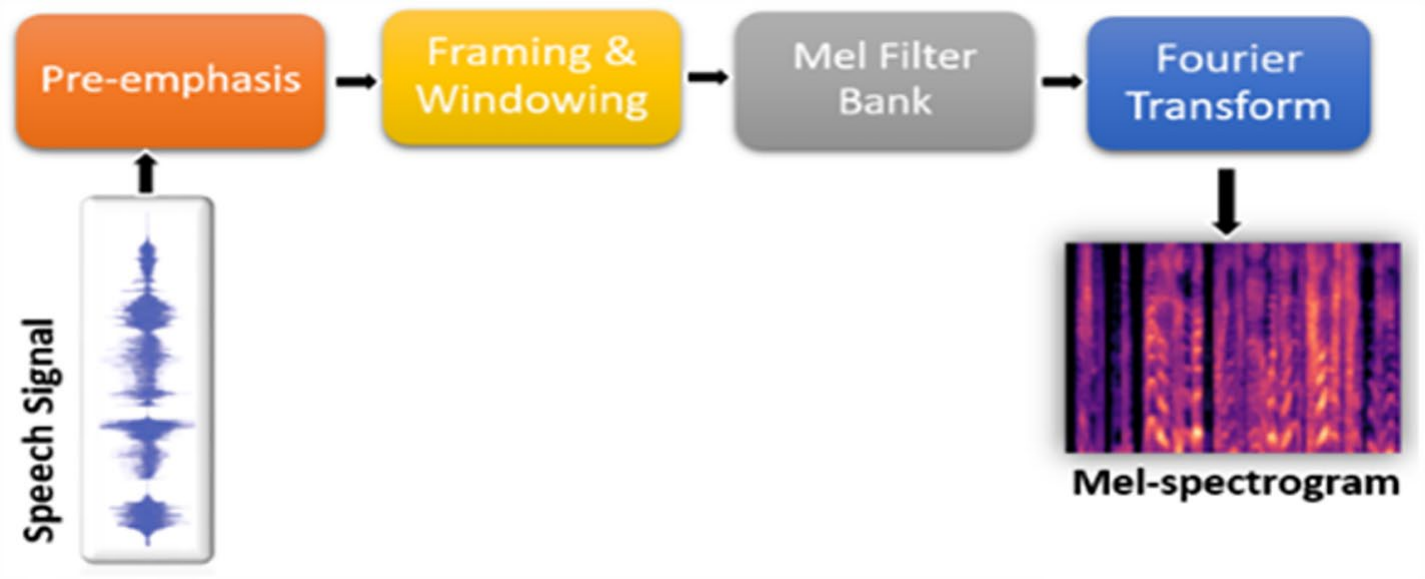
Structure of mel-spectrogram extraction.

Zhang et al.^32^ applied Deep convolutional Neural Network and attention-based network for emotion classification. In their method, a pre-trained DCNN was used as a based model in extracting segment-level features, before the introduction of Bidirectional LSTM for higher-level emotional features. Thereafter, an attention layer was introduced at the top layer of their model, with the utmost focus on features that are relevant to emotion recognition. Their model evaluation achieved UAR of 87.86% and 68.50% respectively on EMODB and IEMOCAP dataset. However, their experiment did not reflect the influence of speech enhancement carried out on raw speech. They augmented the speech corpus used through speed adjustment at varying time-step before the extraction of spectral features was fed into DCNN. Chen et al.^33^ proposed self and global attention mechanism in determining the impact of the attention model on speech emotion classification. Their state-of-the-art approach achieved an accuracy of 85.43% on EMO-DB speech corpus. Their model was built using a sequential network, which requires more computing resources to train. In this paper, two pre-trainned DCNN model are used with attention model and regularized feature selection for SEC. More often than not, many researchers focused on the efficiency of attention mechanism as weight calculator in sequence representation Zhao et al.^34^, however, our proposed model has revealed that the performance of attention-based network is increased when co-join with regularized feature selection for SEC. Nevertheless, this paper concludes with an opportunity for future research in the use of attention mechanism and feature selection to improve the accuracy of classification (Fig. 2).

## Methods and techniques

A general description of the model proposed is given in this section. As a classification problem, speech emotion is categorized rather than dimensional representations^35^. It can be defined as follows, $D = {(X, z)}$, where *X* are input from the acoustic features and z is dimensional output equivalent to the emotion classification. Also, a function $D = {f : X \xrightarrow {} z}$ representing emotional features is to be found before its classification.

**Figure 3:**
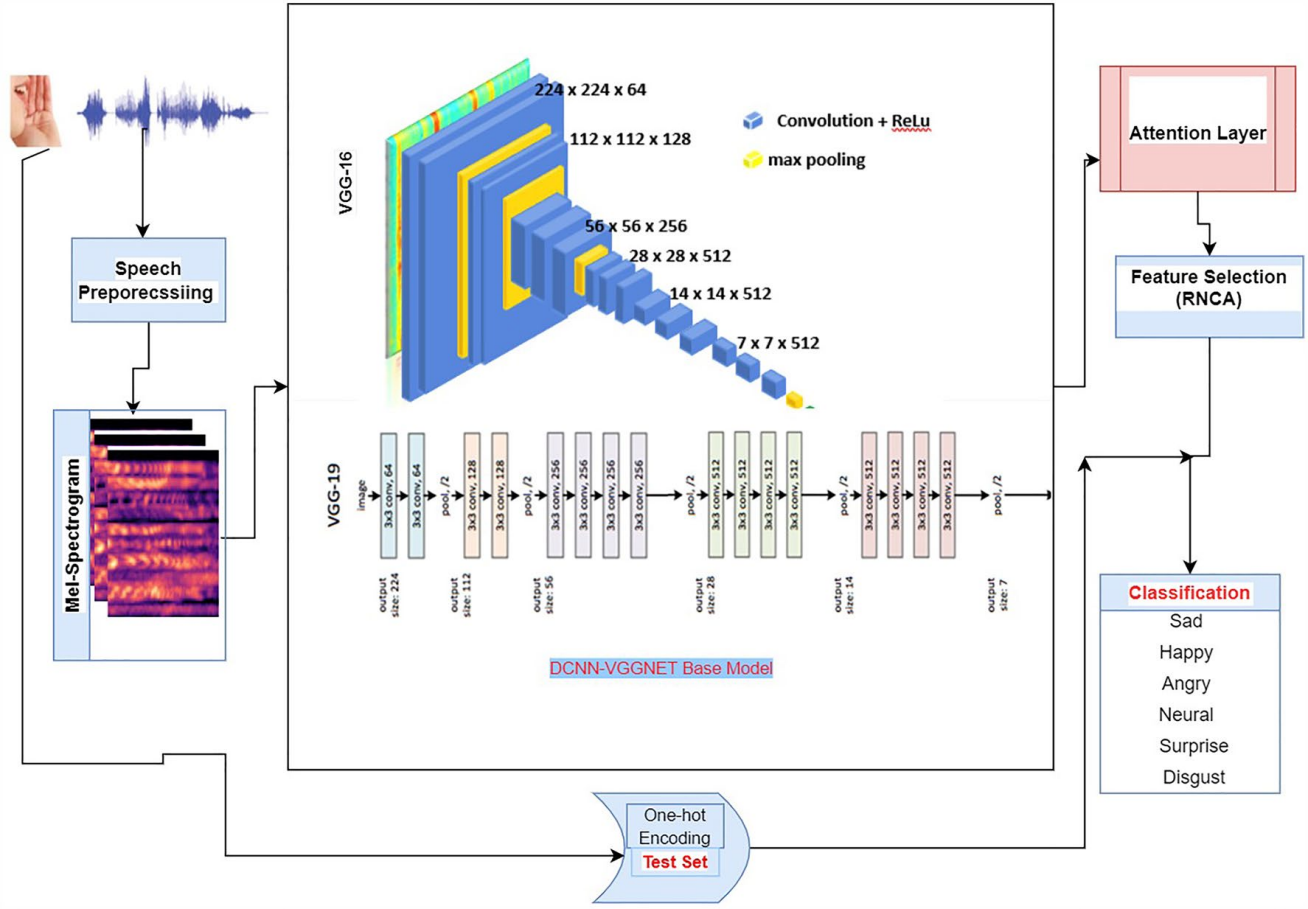
Proposed model architecture.

This study proposed a unique framework for speech emotion classification using attention-based mechanism on pretrained DCNN with regularized feature selection (RNCA) algorithm, as shown in Fig. 3. There are four main phases in our model for speech emotion classification which includes, efficient pre-processing (pre-emphasis) of raw speech from TESS speech corpus, feature learning and extraction, feature selection and emotion classification. As noted in the literature^36^ that the performance of any SEC model rests heavily on dataset pre-processing carried out. In this work, we extracted log mel-spectrogram with three channels (weight, height and input channel) from original speech database containing WAV files. Three channel mel-spectrogram usually comprises of the number of mel-filter banks (in terms of frequency dimension), frame number and the number of channel. The number of channels used for this paper is 3. Three different colours are used to indicate the magnitudes of the Short-Term Fourier Transform (STFT) in a three-channel mel-spectrogram. The low (below 500 Hz-blue), mid (between 500 Hz - 2 kHz-yellow), and high (above 2 kHz-red) frequency ranges of the audio signal are typically represented by the channels, which can offer a more intuitive visual form of the spectral content of the audio signal. The latter is used in this paper. Mel-spectrogram has been widely used^37,38^ in speech-related task, and the reason is not far-fetched from the fact that it’s representation involves time and frequency of speech signals.signal.

At the pre-emphasis stage, the amplification of speech signals (*x*) to high frequency^39^ is performed through a pre-emphasis filter using Eq. (1), where *s*(*t*) represent the speech audio signal before pre-emphasis. We utilized 64 mel-filter banks with 64 frames content window. To obtain the standard frame segment length, we processed $655 ms (10 ms \times 63 + 25)$ fragments, however, a frame segment over 250*ms* has been confirmed^40^ to possess enough paralinguistic information rich enough for emotion classification. The speech signal framing adopted ensures the breaking down of the speech signal into segments of fixed-length. Because the length of human speech varies, framing is required to maintain the size of the voice. The hamming window function of 25*ms* length and 10 ms shift was applied to frames as computed in Eq. (2), where *S* represents the size of the window *w*(*n*). This is illustrated in Fig. 2.

1 $$\begin{aligned}{} & {} y(t) = s(t) - \alpha s(t-1), 0.9 \le \alpha \le 1.0 \end{aligned}$$

2 $$\begin{aligned}{} & {} w(n) = 0.5 - 0.5cos[2\pi \frac{n}{S-1}] 0 \le n = S-1 \end{aligned}$$

The FFT (Fast Fourier Transform) is applied to produce a three channel mel-spectrogram suited as input to our model from raw speech signal with a sample frequency rate of 16*kHz*. This mel-spectrogram can be represented as $M, M \in R^{K \times L \times C}$ where the total number of the filter bank is denoted^32^ by *K* in terms of dimension of the frequency, *L* denotes the length of the segment and the number of channels is *C*.

### Feature extraction

**Figure 4:**
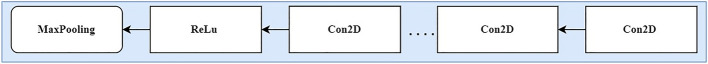
Convolutional layers block diagram.

In this research study, two pre-trained DCNN model serve as our based model (VGG16 and VGG19). We experimented with both pre-trained network on our attention mechanism to establish which one yields better classification performance accuracy after feature selection. We leverage on the weight of these two networks being already trained on ImageNet. Therefore, the convolutional layers comprised of our based model are frozen from training. The input to our model is reshaped from the original $64\times 64 \times 3$ to $224 \times 224\times 3$, as the required input size to the base model of VGGNet.This is achieved using a built-in python library called OpenCV and a bilinear interpolation approach. The base model comprises five convolutional layers with ReLu (Reactivation Linear Unit) activation function for extracting segment-level features from the input mel-spectrogram. A drop-out layer is utilized to prevent overfitting. The output from based model feature extraction is also reshaped to make it suited for the attention layer in extracting high-level emotional features before it is fed into RNCA for eventual feature selection. This is carried out by The block diagram in Fig. 4 depicts the structure of DCNN phase of our model. The pooling layer adopted is max-pooling. This layer performs the function of aggregating the feature sample from the several convolutions of 2D convolutional layers and produces a unified output for the next layer. No fully connected layer was used in the base model.

### Attention layer

Attention mechanism application in computer vision has contributed immensely to the task of image recognition^41^. It mimics the human mode of paying a closer look at what are relevant information that may contribute to their opinion or conclusion on what they see and hear.

In the speech emotion task, the role of the attention network cannot be overlooked, as it carefully concentrates the focus of the model on the frame segment with much emotional content. The attention mechanism lowers the training time^42^ and ensures concentration on features with much emotional information, which can increase model performance. Silent and semi-silent frames are eliminated at the attention layer, as this has a tendency of impairing and distorting the model accuracy. In other words, attention gives insight into the behavioural performance of the deep learning model as it calculates weight from feature representation from the previous layer. The Eq. (3) and (4) indicate how the attention mechanism utilized in this work is computed. Given $X= (x_{1}, x_{2}...x_{n} )$ as the output of features from a convolutional layer.

3 $$\begin{aligned}{} & {} \alpha _{i} = \frac{exp(\mu ^{T}x_i)}{\sum ^{J}_{j=1}exp(\mu ^{T}x_i)} \end{aligned}$$

4 $$\begin{aligned}{} & {} Y = {\sum ^{I}_{j=1}\alpha _{i}x_i} \end{aligned}$$

where alpha $\alpha _{i}$ represents the weight of the attention network, $\mu$ and X are the output of feature representation from the attention layer. At first, the weight of the attention $\alpha _{i}$ is calculated, and it is obtained from Eq. (3) (softmax function) through the training process. *Y* is got from the weighted sum of *X*, as deeper features at the utterance level. The attention mechanism has proven to be of tremendous help in generating more distinctive features for SEC. The attention layer is responsible for dynamically highlighting and weighting various input feature components according to their applicability to the emotion recognition task. The power of the model to successfully learn and represent the attention weights depends on the number of neurons in the attention layer. We used 128 neurons, increasing our model’s capacity for capturing fine-grained feature importance while minimizing complexity.

### Regularized neighbourhood component analysis (RNCA) feature selection

The RNCA feature selection mechanism is a specific class of feature weighting approach that carries out its operation by learning feature weight and maximizing the leave-one-out (LOO) accuracy of classification over sample data^43^. The LOO provides an unbiased estimate of a deep learning model performance. RNCA works by assessing the vector weight *w* that corresponds to the feature vector $x_i$ through the optimization of a classifier that is based on the nearest neighbour scheme. It has a mechanism for controlling complexity and preventing overfitting on the density estimation. RNCA adopts a framework of selecting a certain reference sample called $x_j$ for the sample $x_i$ from all emotion feature samples randomly. However, the probability of the selected feature $(P_{ij})$ to $x_j$ rest heavily on the distance $D_w$ that exists between two samples. This distance can be computed^44^ as in Eq. (5) below:

5 $$\begin{aligned} D_{w}(x_i, x_j) = \sum _{m=-1}^{r} w{^2}{_m}|x_{im} - x_{jm}| \end{aligned}$$

Where *mth* the feature’s weight is denoted by $w_m$. A kernel function k established the relation $P_{ij}$ and Dw on the condition that the smaller the Dw the larger the values of k. The likelihood $P_{ij}$ and kernel function *k* can be computed for Eqs. 6 and 7 respectfully as below

6 $$\begin{aligned}{} & {} P_{ij} = \frac{k(D_w(x_i, x_j)}{\sum ^{n}_{j=1, j\not = i}k(D_w(x_i, x_j)} \end{aligned}$$

7 $$\begin{aligned}{} & {} k(z) = exp -\frac{z}{\sigma } \end{aligned}$$

where the kernel width is represented by $\sigma$ that influences the likelihood that a reference point selected will be $x_j$ sample. Therefore, the likelihood of correctly classifying $x_i$ can be computed from Eq. (8).

8 $$\begin{aligned} P_i = \sum ^{n}_{j=1, j\not = i} P_{ij}Y_{ij} \end{aligned}$$

Where $y_{ij}$ can only indicate one if both $y_i$ and $y_j$ are equal to each other. The average LOO accuracy of classification is the sum of all $P_i$ of all the samples divided by the total number of samples, as indicated in Eq. (9). This equation can be termed as the objective function that required maximization. Nevertheless, the objective function defined above is not insulated from overfitting, which calls for the introduction of a parameter $\lambda$ termed regularizer to prevent overfitting. The modified objective function that represents RNCA can be defined as

9 $$\begin{aligned} Obj. (A) = \sum ^{n}_{i=1} P_{i} - \lambda \sum ^{r}_{m=1} w{^2}{_m} \end{aligned}$$

**Figure 5:**
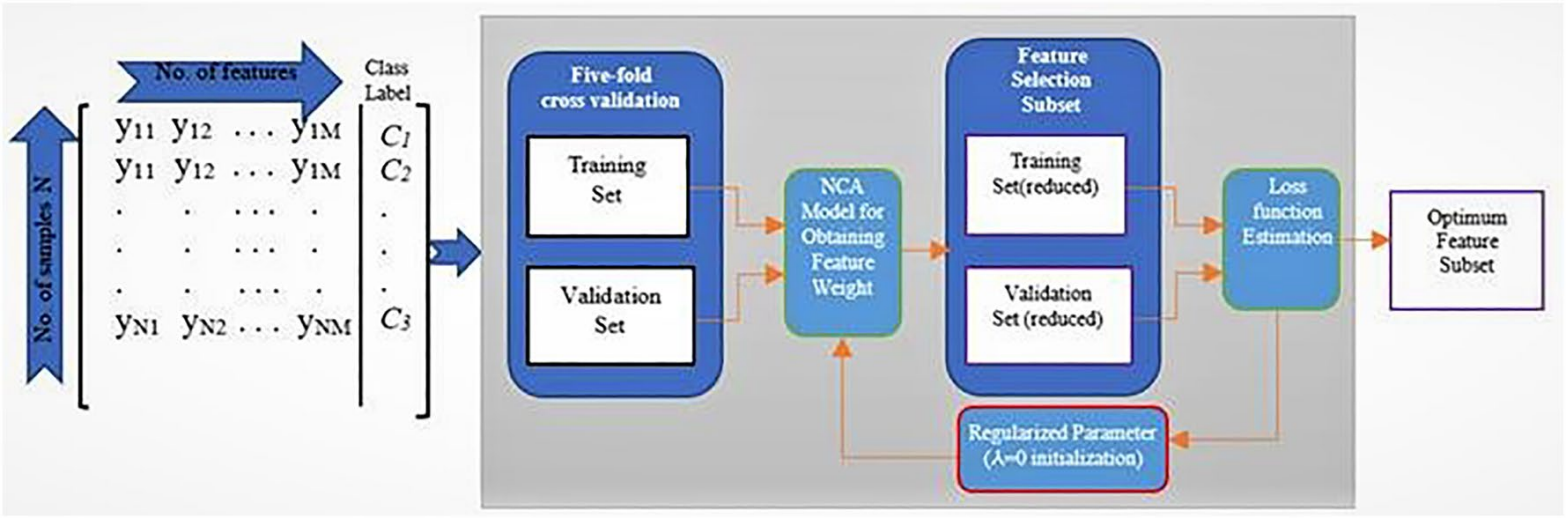
RNCA framework.

The RNCA algorithm adopted in this work operates on the output from the attention layer of our model to aid feature selection, therefore, it is essential to evaluate generalization error (Eq. 10) to properly fine-tune the regularization parameter $\lambda$ to obtain a minimized classification loss.

10 $$\begin{aligned} Err = \frac{1}{n}\sum ^{N}_{i=1} I(k_{i}\not = t_{i}) \end{aligned}$$

where the predicted label is represented by $k_i$ and $t_i$ denotes the real label of the feature sample. The RNCA feature selection technique is diagrammatically shown in Fig. 5.

### Emotion classification

In this study, three primitive classifiers were utilized in carrying out the classification of emotion. The classifiers take their simplified input from the output of the feature selection layer of our model after feature extraction. The essence of employing three different classifiers is to ensure the robustness of the entire model and aid the analysis of the result. Multi-layer perceptron (MLP) classifier is first introduced. As a feedforward network-based classifier^6^, a set of suitable outputs are mapped from a set of input datasets by this feedforward artificial neural network model. An MLP is made up of several layers, each of which is completely connected to the one before it. Except for the nodes in the input layer, the nodes of the layers represent neurons with nonlinear activation functions.

Secondly, we also utilized a support vector machine (SVM). An SVM operates as a discriminative classifier, well-defined by dividing hyperplane. It fits into supervised and unsupervised machine-learning tasks. For instance, given a set of selected features (or data), the algorithm outputs an optimal hyperplane that classifies new samples. In two-dimensional space, this hyperplane is a line dividing a plane into two parts, wherein each class lay on either side^45^. Besides, SVM can effectively handle multiclass problems as it is obtainable with emotion classification. One distinguishable function of SVM is that it selects a hyper-plane with a large margin, reducing the likelihood of miss-classification and its low sensitivity to outliers.

Lastly, Random Forest (RF) was also employed as the third classifier. RF is a meta-estimator that employs averaging to increase classification accuracy and reduce overfitting after fitting several decision tree classifiers to different emotional feature subsamples. Random forest possesses an inbuilt mechanism for managing class imbalance, and this has given it an edge over other classifiers.

## Experimental results and discussion

### Dataset

In this study, we benchmarked our experiment on one of the publicly available datasets named Toronto English Speech Set (TESS). In 2010, at Northwestern University’s Auditory Laboratory, TESS speech samples were recorded^46^. During the spontaneous event, two actresses were asked to recite a handful of the 200 words, and their voices were recorded, resulting in a complete collection of 2800 speech utterances. Seven different emotions comprise happy, angry, fear, disgust, pleasant, surprise, sad and neutral were observed in the scene.

### Experimental configuration

In this study, the experiment was carried out using a 64-bit operating system, an Intel Core i7 processor, 8 GB of RAM, and a Python 3.9 environment. Deep learning software and additional third-party libraries (including Tensorflow, Numpy, and audio processing) were also utilized. The audio sample first needed to be pre-processed because the input layer for our model has to be in 224 x 224 x 3. To meet the requirements of the model, the voice signal has to be scaled and transformed into a log-mel spectrogram. The FFT technique was used to separate the mel-spectrogram feature from the original audio data. The dataset is then sectionalized into a training set and a testing set (80%:20%). Both the exam and practice sets’ data were normalized to pixels.

### Implementation parameters

In implementing our model and compilation of the network, we utilized the Adams optimizer with a learning rate set to 5e-5 notation. One-hot encoding technique was used in vectorizing the label. It ensures that the data point is binarized. We adopted sparse categorical cross entropy for the loss function. To actualize the objective of increasing accuracy, we initialize our model set up with 100 epochs and 16 batch size, however the result of our training after 25 epochs yielded optimum accuracy. We utilized a custom-early stopping mechanism to monitor (checkpoint) the loss and accuracy value to prevent overfitting, and the corresponding curve was obtained as well.

#### Experimental Results

**Figure 6:**
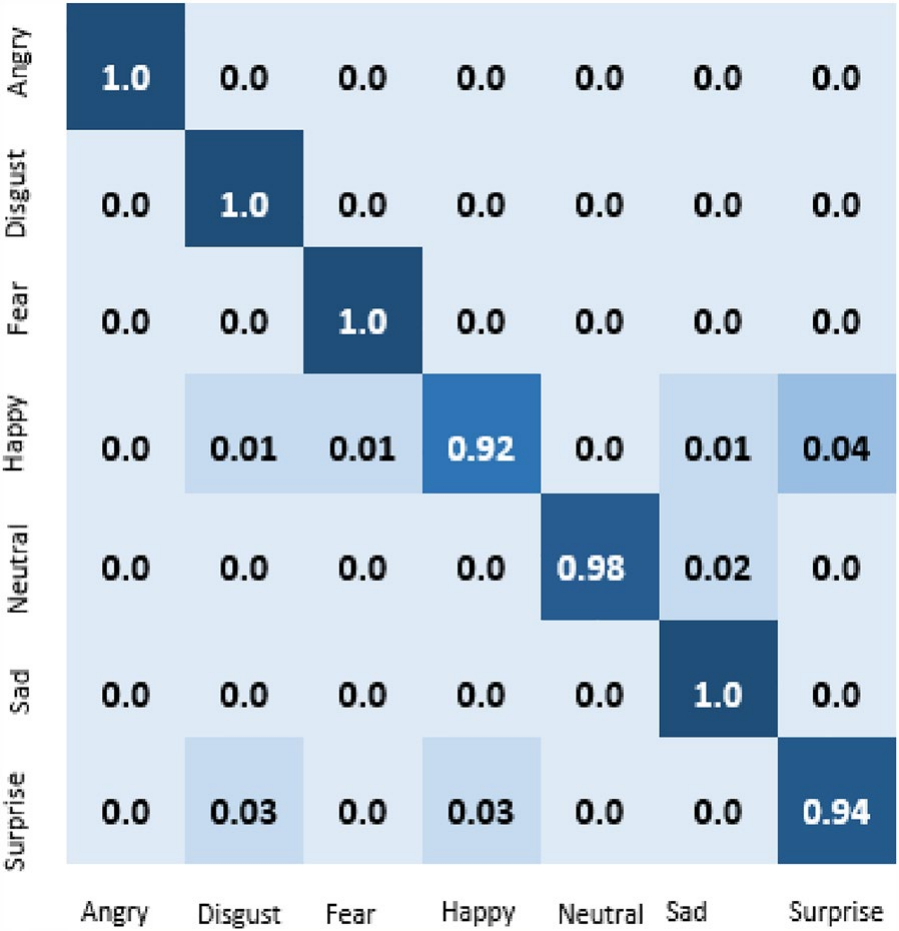
Attention-based Vgg16+RNCA+RF.

**Figure 7:**
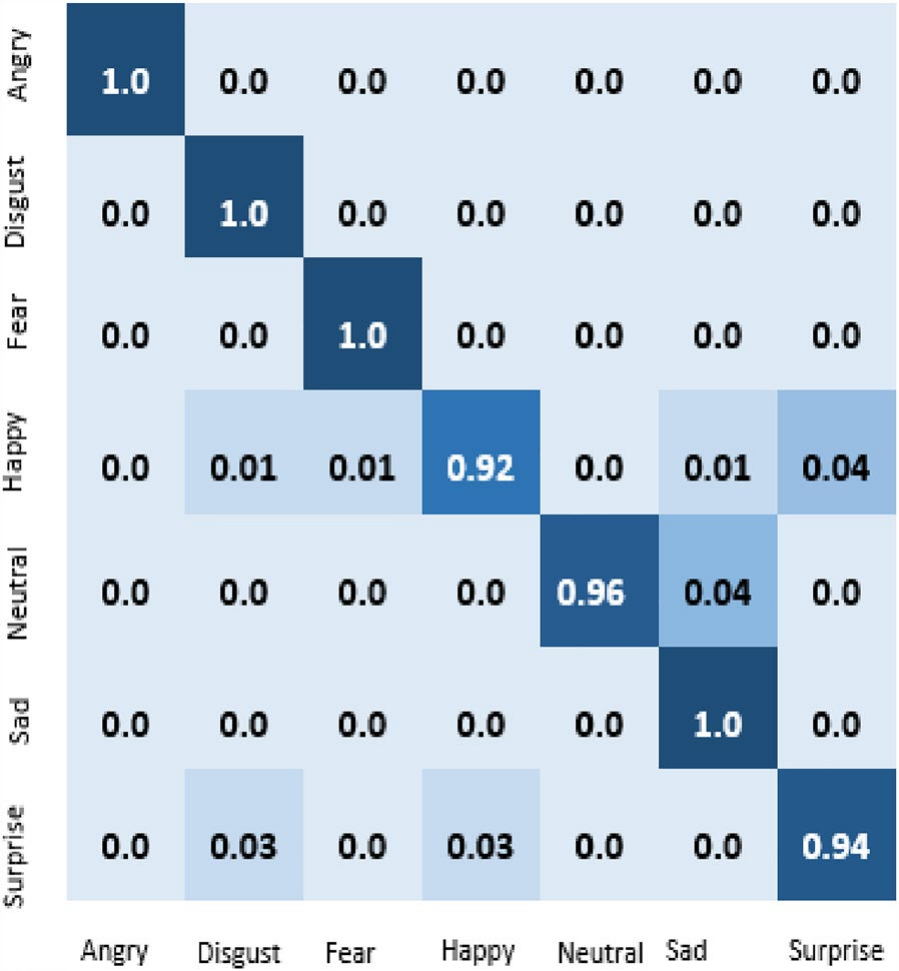
Attention-based Vgg16+RNCA+MLP.

**Figure 8:**
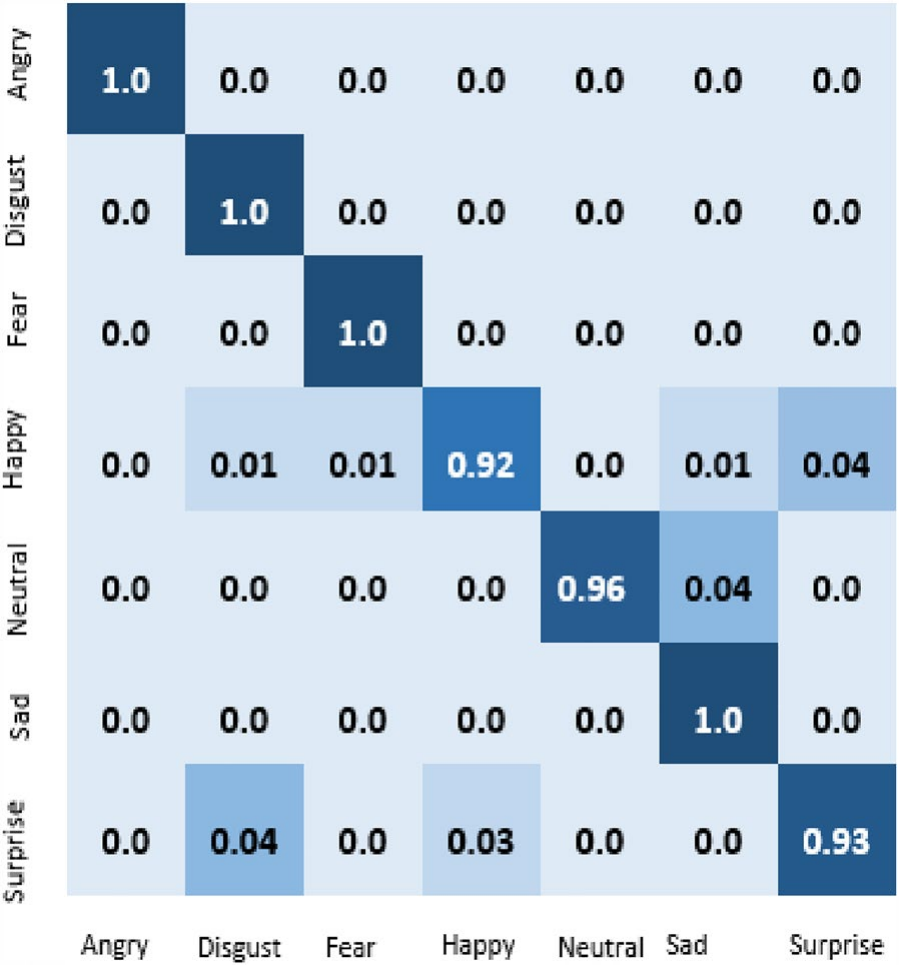
Attention-based Vgg16+RNCA+SVM.

**Figure 9:**
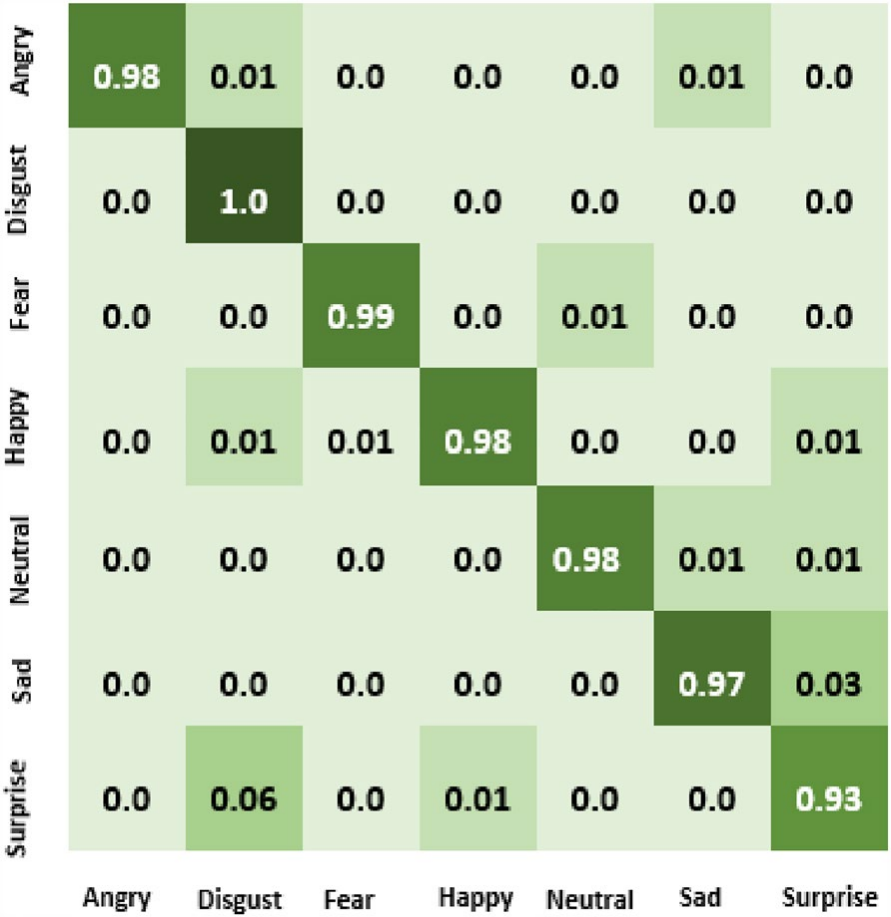
Attention-based Vgg19+RNCA+RF.

**Figure 10:**
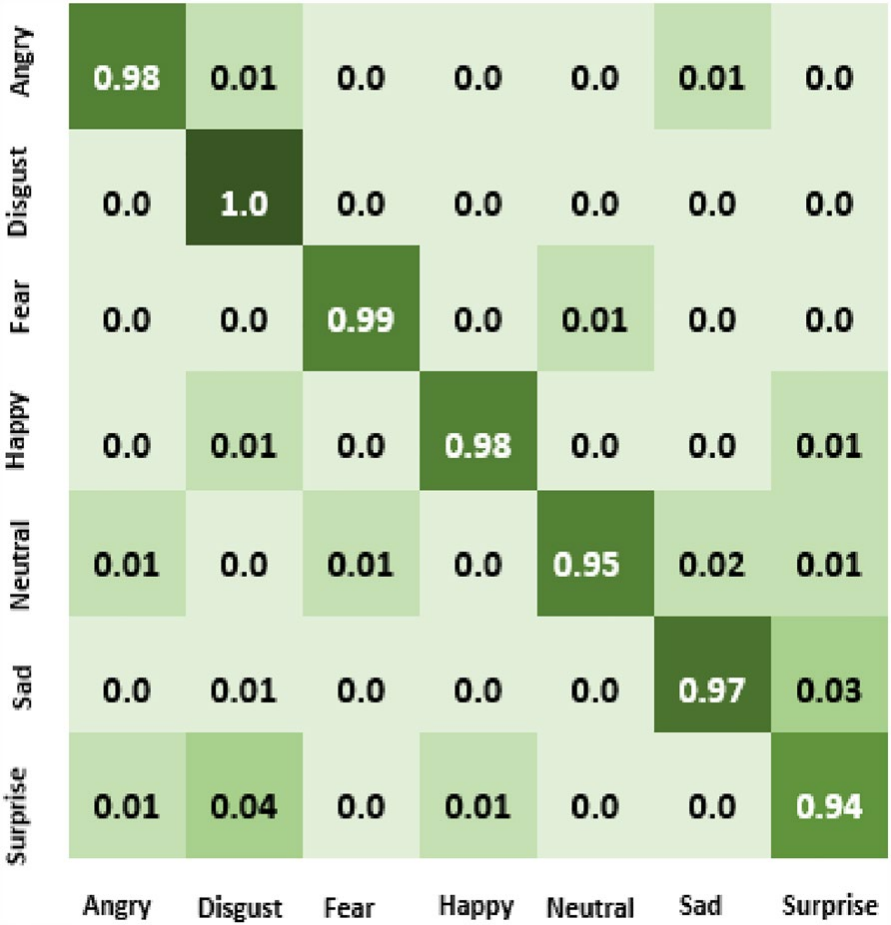
Attention-based Vgg19+RNCA+MLP.

**Figure 11:**
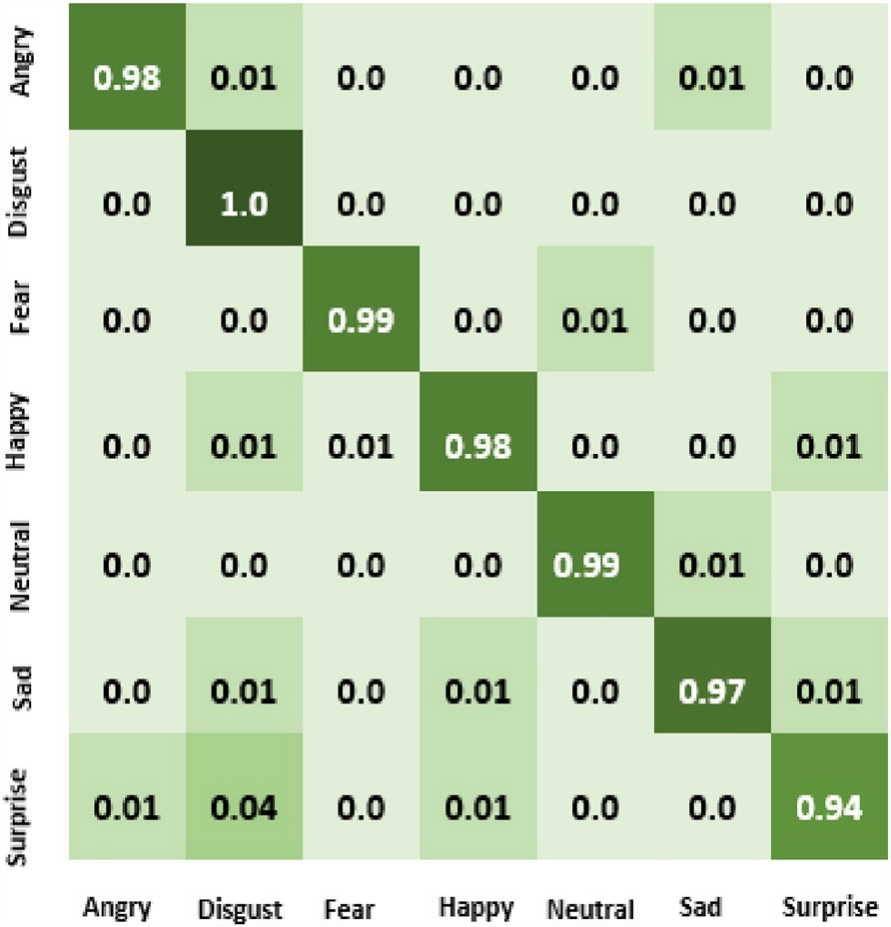
Attention-based Vgg19+RNCA+SVM.

**Figure 12:**
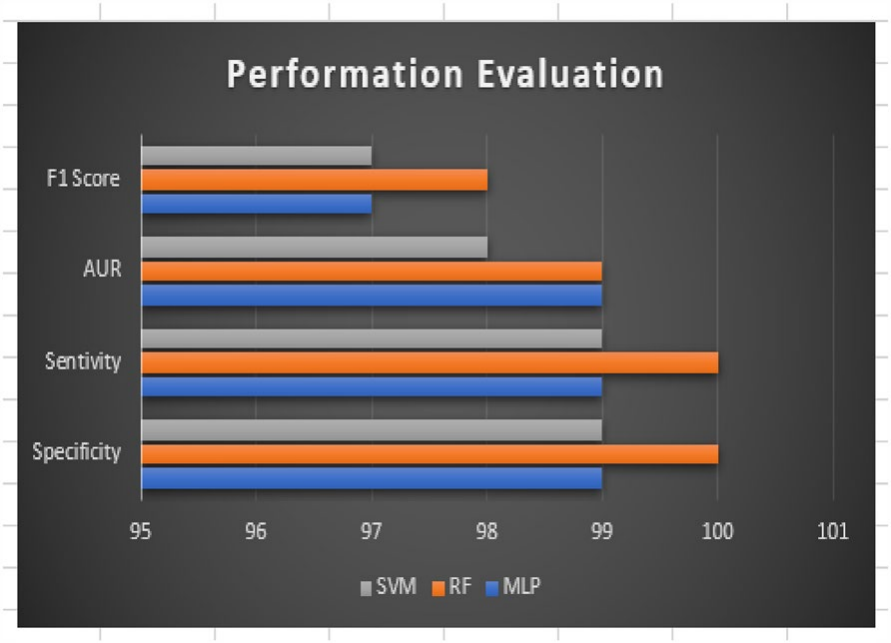
Performance chart with 4 metrics and 3 classifiers.

The result of our experiment using attention-based networks and regularized feature selection with three classifiers are presented in this section. For the first experiment where the Vgg16 pre-trained network was utilized, the confusion matrix of emotion classification is shown in Figs. 6, 7, 8, 9. We observed that the attention network of our model achieved the highest accuracy (97.8%) of recognition with the RF classifier compared to the other classifiers (SVM:97.4% and MLP: 97.6%). From the figures, the emotional class of angry, disgust, fear and sad accuracy reach 100% with the attention-based network and RF, SVM and MLP. The Neural emotion class got 98% the highest accuracy of recognition with the RF classifier, while 94% best accuracy was obtained on surprise emotion from Figs. 6 and 7 respectively (Figs. 10 and 11). The performance evaluation chart in Fig. 12 shows other evaluation metrics (specificity, sensitivity, F1-score and unweighted average recall) used to establish the robustness of our model. The two experiments are captured on the chart.

In our second experiment, the pre-trained model used was Vgg19 before the attention layer was added. The result generated is shown in Figs. 9, 10, 11. Disgust emotion carries the highest classification accuracy of 100% from the three classifiers, while surprise emotion has the least classification accuracy of 93%. Neutral emotion differs in accuracy from the three classifiers, its optimum accuracy is at 99% with the SVM classifier. The overall model classification accuracy obtained from the second experiment is 97.5%. This is low compared to the previous experiment where vgg16 was used as the convolutional layer, however, the impact of the attention network for the extraction of emotionally related features combined with regularized feature selection has improved the classification accuracy of speech emotion.

**Figure 13:**
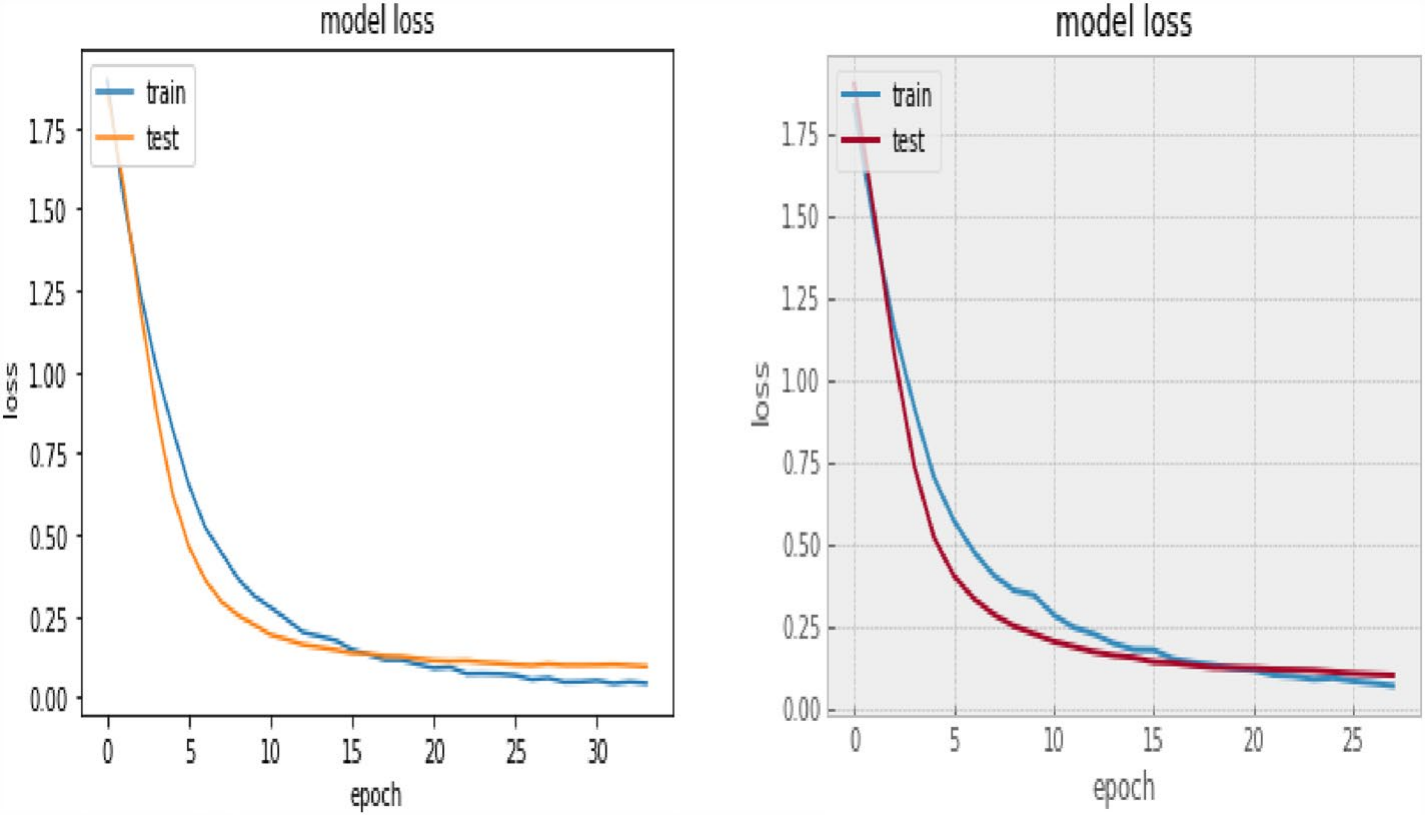
Model loss curve.

**Figure 14:**
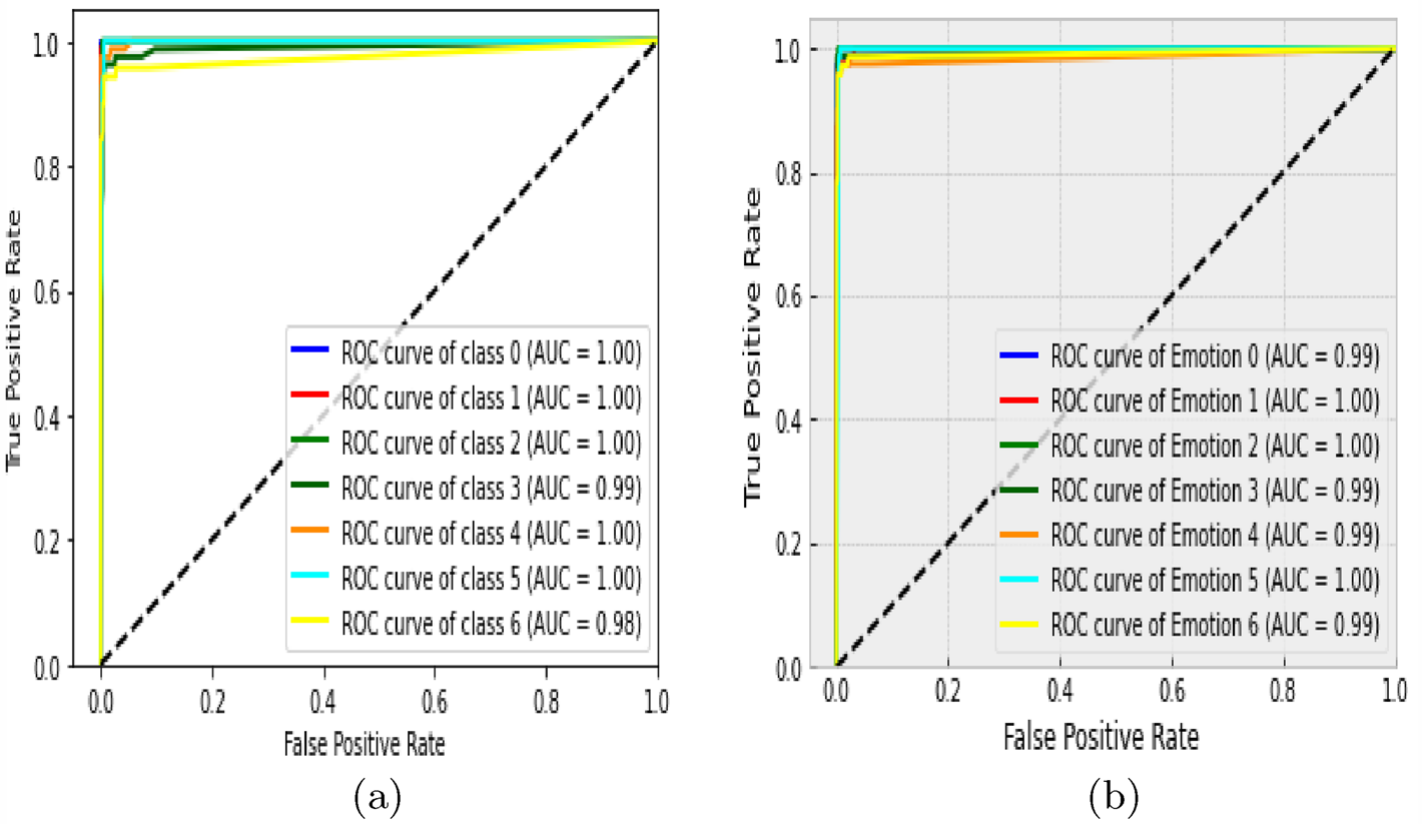
ROC curve.

Besides the accuracy obtained through the confusion matrix, the model loss and ROC (Return of Characteristics) curves in Figs. 13 and 14 further testify to the performance of our model in this paper. The loss value from the curve is relatively low, indicating that our model has prevented overfitting. The loss curve decreases over time as our model improved. Also, the loss curve shows a smooth convergence which is a further indication that our model prediction is accurate to an acceptable level. The low initial loss value with respect to the convergence point as shown confirmed the reduction in model complexity and training time. The ROC curve shows the seven categories of emotion as indicated in Table 1 below with the area under the curve (AUC) which demonstrates the performance average across all potential emotion classification thresholds. The diagonal dotted line is the threshold. The closeness of the curve to the top left-hand corner for the seven emotional classes indicates a high True Positive Rate (TPR) and low False Positive Rate (FPR). The least AUC score recorded is 0.98, an evidence of the good performance of our model on emotion classification.

In this work, the Mel-spectrogram was used to extract the input feature, producing a feature vector with a dimensionality of 40 (mel frequency bins). These features record important details about the speech signal’s spectral composition and temporal dynamics. The feature space was high dimensional, so feature selection was used to lower the dimensionality and concentrate on the most useful features for the task. The feature selection algorithm assessed each feature’s relevance based on how it contributed to the performance of emotion recognition, taking into account measures like mutual information and feature importance scores, thereby increasing the model’s efficiency, lowering the amount of computing power required, and improving the interpretability of the learned representations. Our experiments’ findings showed that feature selection significantly enhanced the speech emotion recognition model’s performance, resulting in an increase in accuracy of 3.7%, underscoring the significance of feature selection in improving the model’s discriminative power for emotion recognition tasks.

**Table 1:** Return of Characteristics Description.

Emotion	Class	AUC score (%)
Angry	0	100
Disgust	1	100
Fear	2	100
Happy	3	99
Neutral	4	100
Sad	5	100
Surprise	6	98

#### Performance Comparison

Additionally, our proposed model in this study was compared with other work carried out by others benchmarked on the same speech dataset, as indicated in Table 2. We also carried out a comparative analysis of our proposed model without the attention layer, RNCA, and with the attention mechanism and RNCA feature selection as shown in Table 3.

**Table 2:** Comparison of our proposed with other methods.

Publication	Model	Dataset	Accuracy (%)
2017^47^	MFCC+SVM	TESS	96.00
2018^48^	DNN+GRU	TESS	95.82
2019^49^	MFCC+CNN	TESS	81.00
2021^50^	IMF+SVM+KNN	TESS	93.30
2022^51^	DNN+NCA+MLP	TESS	96.10
Proposed	Attention-based DCNN+RNCA+RF	TESS	97.8

**Table 3:** Emotional level comparison of the significance of attention and feature selection.

Emotion	Without attention(%)	Without RNCA(%)	Attention + RNCA(%)
Angry	98	100	96.00
Sad	91	91	95.82
Surprise	86	89	81.00
Happy	98	100	93.30
Fear	96	100	96.10
Neutral	95	98	96.10
Disgust	94	100	96.10
Average	94.00	96.85	97.71

In terms of accuracy, reduction of complexity and prevention of overfitting, our method surpasses other methods^47–51^ utilized for speech emotion classification or recognition.

## Conclusion

In this study, we proposed a SEC system using an attention-based network and regularized feature selection. First and foremost, we extracted the mel-spectrogram from the TESS dataset used for this study. This was carried out, after extensive speech processing and analysis, to feed (input layer) our model with appropriate features for enhanced feature extraction in the subsequent layers. A pre-trained DCNN base model was adopted for our attention network to extract local features, while the attention layer deals with emotionally rich features (global features) which ultimately reduces misclassification to the barest minimum. The core principle of the attention network is to estimate feature weight. In our attempt to increase the efficiency of our model, a regularized feature selection is introduced after the attention layer to actualize optimum results. The feature selection aided the attention mechanism to focus more on salient features. Thereafter, three classifiers were fed with selected emotional features with RNCA, for the classification of emotion.

After a comparison of the result of our experiments, an attention-based DCNN+RNCA+RF model for speech emotion classification was proposed. The experimental result attained the optimum accuracy of 97.8% on the TESS dataset. Seven classes of emotions comprised of anger, sad, happy, fear, neutral, disgust and surprise that reflect human major emotions were accurately classified. Besides, by contrasting our proposed model in this study with other methods that have recently been put forward, obviously, our model outperforms many of them in speech emotion classification tasks.

Moreover, the computational cost peculiar to most deep learning tasks is prevented in this study, simply because our based model for the attention network requires no training and the total number of trainable parameters has been reduced to the barest minimum (101,480) out of the total parameters of 14,017,704. The number of floating-point operations per seconds(FLOPs), and the model’s (size of 98MB) memory requirement have been reduced to minimize complexity because the top layer of the VGGNet has been frozen. The average time taken for each emotional utterance to be classified by the proposed model is 0.12. However, though, the result obtained from this study has undoubtedly provided some insight for researchers on the application of attention mechanism with feature selection for SEC tasks, we recommend that future work can be carried out using a sequential network, more pre-trained based network, low-level features and introduction of other speech emotion dataset.

## Data Availability

Benchmarked publicly available dataset, Toronto English Speech Set (TESS) is used.
